# New insights into the phylogenetics and population structure of the prairie falcon (*Falco mexicanus*)

**DOI:** 10.1186/s12864-018-4615-z

**Published:** 2018-04-04

**Authors:** Jacqueline M. Doyle, Douglas A. Bell, Peter H. Bloom, Gavin Emmons, Amy Fesnock, Todd E. Katzner, Larry LaPré, Kolbe Leonard, Phillip SanMiguel, Rick Westerman, J. Andrew DeWoody

**Affiliations:** 1 Department of Biological Sciences, Towson University, 8000 York Rd, Baltimore, MD 21212 USA; 2 Department of Forestry and Natural Resources, Purdue University, 715 W. State Street, West Lafayette, IN 47907 USA; 3 East Bay Regional Park District, 2950 Peralta Oaks Court, Oakland, CA 94605 USA; 4 Department of Ornithology and Mammalogy, California Academy of Sciences, 55 Concourse Drive, Golden Gate Park, San Francisco, CA 94118 USA; 5 Bloom Research Inc., 1820 S. Dunsmuir, Los Angeles, CA 90019 USA; 6 National Park Service, Pinnacles National Park, 5000 Highway 146, Paicines, CA 95043 USA; 7 California State Office, Bureau of Land Management, 2800 Cottage Way, Suite W-1928, Sacramento, CA 95825 USA; 8 U.S. Geological Survey, Forest and Rangeland Ecosystem Science Center, 970 Lusk Street, Boise, ID 83706 USA; 9 Bureau of Land Management, California Desert District, 22835 Calle San Juan De Los Lagos, Moreno Valley, CA 92553 USA; 10 Department of Computer and Information Sciences, Towson University, 8000 York Rd, Baltimore, MD 21212 USA; 11 Department of Horticulture and Landscape Architecture, Purdue University, West Lafayette, IN 47907 USA; 12 Department of Biological Sciences, Purdue University, 915 W. State Street, West Lafayette, IN 47907 USA

**Keywords:** Panmixia, Hierofalcons, SNP genotyping, Avian genome assembly, Molecular sexing, Repeatability, Selection, Phylogenomics

## Abstract

**Background:**

Management requires a robust understanding of between- and within-species genetic variability, however such data are still lacking in many species. For example, although multiple population genetics studies of the peregrine falcon (*Falco peregrinus*) have been conducted, no similar studies have been done of the closely-related prairie falcon (*F. mexicanus*) and it is unclear how much genetic variation and population structure exists across the species’ range. Furthermore, the phylogenetic relationship of *F. mexicanus* relative to other falcon species is contested. We utilized a genomics approach (i.e., genome sequencing and assembly followed by single nucleotide polymorphism genotyping) to rapidly address these gaps in knowledge.

**Results:**

We sequenced the genome of a single female prairie falcon and generated a 1.17 Gb (gigabases) draft genome assembly. We generated maximum likelihood phylogenetic trees using complete mitochondrial genomes as well as nuclear protein-coding genes. This process provided evidence that *F. mexicanus* is an outgroup to the clade that includes the peregrine falcon and members of the subgenus Hierofalco. We annotated > 16,000 genes and almost 600,000 high-quality single nucleotide polymorphisms (SNPs) in the nuclear genome, providing the raw material for a SNP assay design featuring > 140 gene-associated markers and a molecular-sexing marker. We subsequently genotyped ~ 100 individuals from California (including the San Francisco East Bay Area, Pinnacles National Park and the Mojave Desert) and Idaho (Snake River Birds of Prey National Conservation Area). We tested for population structure and found evidence that individuals sampled in California and Idaho represent a single panmictic population.

**Conclusions:**

Our study illustrates how genomic resources can rapidly shed light on genetic variability in understudied species and resolve phylogenetic relationships. Furthermore, we found evidence of a single, randomly mating population of prairie falcons across our sampling locations. Prairie falcons are highly mobile and relatively rare long-distance dispersal events may promote gene flow throughout the range. As such, California’s prairie falcons might be managed as a single population, indicating that management actions undertaken to benefit the species at the local level have the potential to influence the species as a whole.

**Electronic supplementary material:**

The online version of this article (10.1186/s12864-018-4615-z) contains supplementary material, which is available to authorized users.

## Background

Management of species occurs at multiple scales, requiring a robust understanding of between- and within-species genetic variability. For example, identification of cryptic species (e.g., giraffes [1]) and subspecies (e.g., chimpanzees [2]) allows resources to be allocated to previously unrecognized lineages. Furthermore, an understanding of “evolutionary distinctiveness” (i.e., how isolated a species is within a phylogeny) can result in unique lineages being prioritized for protection [3, 4]. At the population level, an understanding of within-species structure [5–7] and adaptive genetic differentiation [8–10] allows biologists to identify evolutionarily distinct and/or demographically independent population units of conservation interest [11–13] and assign conservation priority.

However, the extent to which genetic variability, population structure and phylogenetic relationships are documented varies drastically across species. For example, multiple population genetics studies of the peregrine falcon (*F. peregrinus*) have been conducted throughout the species range (e.g., [14–18]) but no similar studies have been done of the closely-related prairie falcon (*F. mexicanus*). Genomic tools (e.g., whole genome sequencing and SNP genotyping arrays) can rapidly provide insight in species whose genetics have been historically under-studied. High-throughput sequencing and/or SNP assays allow hundreds or thousands of loci to be quickly and affordably genotyped. Larger suites of markers produce more accurate assessments of genome-wide heterozygosity and lead to statistically rigorous phylogenetic reconstructions [19].

Herein, we describe the genomic approaches taken to describe genetic diversity in the prairie falcon (*Falco mexicanus*) relative to other species and across populations. The prairie falcon range extends from Canada (e.g., British Columbia and Alberta) into the western United States (Washington, Idaho and Montana) south to California, Arizona, New Mexico and ultimately into Mexico [20]. *F. mexicanus* nests on cliffs and thrives in diverse habitats throughout western North America – from desert and shrub-steppe to grassland and oak-savannah-chaparral [21, 22]. Prairie falcons prefer to feed on ground squirrels even when they are rare relative to other prey species, which include passerines, reptiles, insects and other small mammals [21, 23].

*F. mexicanus* populations can be adversely affected by anthropogenic development [24–26]. Humans indirectly affect prairie falcons by altering natural habitats and decreasing the availability of prey, foraging opportunities or nesting sites. For example, Steenhof et al. [27] argued that spatial patterns of abundance and productivity stemmed from decreased foraging opportunities likely associated with interactions among military training activities, fire and grazing intensity in the Morley Nelson Snake River Birds of Prey National Conservation Area in Idaho. Collisions with wind turbines, in turn, represent a direct threat to prairie falcons [28]. Across the *F. mexicanus* range, population numbers as indicated by migration data and Western Breeding Bird Survey data appear stable or increasing [29]. However, Christmas Bird Counts decreased linearly between 1977 and 2001 ([29], but see [30]) and declines of occupied nesting territories have been noted locally (e.g., San Francisco East Bay Area; unpublished observations, DA Bell).

An understanding of the underlying genetic variation present in western *F. mexicanus* is integral to managing the species, as variability is a requirement for species to respond to changing environments and selection pressures [31–33]. Furthermore, it is unclear whether prairie falcons in the western United States represent a randomly mating population or genetically distinct units that should be managed separately. To evaluate the current status of the prairie falcon, we developed a draft genome sequence and SNP assay, with the aim of better understanding genetic variability, population structure and adaptive genetic differentiation throughout California and Idaho. Of particular interest is the extent to which gene flow exists amongst prairie falcons nesting in three separate geographic regions in California: the San Francisco East Bay Area, Pinnacles National Park and the Mojave Desert. These areas are undergoing rapid development or are subject to extensive land-use changes, potentially threatening local nesting *F. mexicanus*.

In addition to this work, we take advantage of our sequencing approach to explore the phylogenetic relationship of the prairie falcon to other falcon species. Historically, the prairie falcon was clustered into the subgenus Hierofalco, which includes the lanner falcon (*F. biarmicus*), saker falcon (*F. cherrug*), lager falcon (*F. jugger*) and gyrfalcon (*F. rusticolus*), based on ecological and morphological similarities [34, 35]. Subsequent phylogenies generated from sequencing data have indicated that *F. mexicanus* is more closely related to *F. peregrinus* than to the hierofalcons. However, branching patterns differ amongst these phylogenies which are based on relatively small portions of the mitochondrial genome [36–39]. Accordingly, we use nuclear protein-coding genes and the complete mitochondrial DNA sequence of the prairie falcon, described herein, to revisit the phylogeny of *Falco*.

## Methods

### Nuclear genome sequencing, assembly and annotation

A female prairie falcon was captured in Siskiyou County, California on 7 June 2014. Two drops of blood were collected via venipuncture of the brachial vein and preserved in lysis buffer (100 mM tris hydrochloric acid, 100 mM ethylenediaminetetraacetic acid, 10 mM sodium chloride, 2% sodium dodecyl sulfate). We extracted DNA (deoxyribonucleic acid) using potassium acetate extraction [40].

We conducted one lane each of paired-end (PE; read length: 100 bp [base pairs]; average fragment length: 568 bp) and mate-paired (MP; read length: 100 bp; average fragment length: 2210 bp) sequencing using an Illumina HiSeq2000 (Table 1). Trimmomatic [41] was used to remove adaptors, discard short reads (< 30 bp), and trim poor quality bases (Illumina Q-value ≤20) from both 5′ and 3′ ends of raw sequence reads. The process described above is appropriate given that the program used for genome assembly accounts for the presence of low quality nucleotides and overly stringent trimming decreases assembly quality [42]. Similarly, GATK (the pipeline used for SNP discovery, see below) requires only the removal of adaptor sequences and subsequently addresses sequencing errors and duplicate reads internally [43, 44]. Fragment lengths and insert sizes were estimated using Picard (http://broadinstitute.github.io/picard).

**Table 1:** Summary statistics for prairie falcon (*Falco mexicanus*) paired-end (PE), mate-paired (MP) and long read (LR) libraries

Library	Mean fragment length (bp)	Inferred insert size (bp)	Raw data		Following quality control	
			Total data (Gb)	Total reads	Total data (Gb)	Total reads
PE	568	368	41.1	407,214,416	37.9	385,316,766
MP	2210	2010	35.0	346,792,322	25.7	278,882,670
LR			50.9	514,493,678	50.5	510,447,548

We additionally generated Illumina TruSeq Synthetic Long Reads (LRs; [45, 46]). To complete the LR sequencing process, we 1) selected 384 genomic DNA fragments 10 kb (kilobases) in length, each of which underwent additional fragmentation, tagging and indexing in an individual well, 2) pooled and purified genomic material from all 384 wells and 3) sequenced the libraries on a single lane using an Illumina HiSeq2000. We again removed adaptors, discarded short reads and trimmed poor quality bases (see above) from the 100 bp reads and the program SPAdes 3.1.1 [47] was used to assemble sequenced fragments into ~ 10 kb LRs.

We used ABySS 1.5.2 [48] to conduct several preliminary assemblies of PE and LR reads, using kmer lengths ranging from 35 to 90. We determined that kmer lengths of 50 or 60 produced the best assemblies by considering both N50 values and the length of the longest scaffold. Final draft assemblies were completed by assembling PE reads into contigs before using both LR and MP reads in the scaffolding step, considering kmer lengths of just 50 and 60. The best draft assembly was chosen by considering both N50 values and the length of the longest scaffold. CEGMA 2.5 [49] was used to identify core eukaryotic genes present in the draft assembly.

We used the MAKER 2.28 pipeline to annotate the draft prairie falcon genome as in Doyle et al. [50]. Briefly, RepeatMasker [51] identified and masked stretches of repetitive DNA, while SNAP [52] and AUGUSTUS [53] were used to generate *ab initio* gene predictions. Gene predictions were subsequently elevated to gene annotations if expressed sequence tag (EST), protein or InterProScan evidence supported the prediction. *Falco cherrug* EST sequences were assembled using Trinity as described in Doyle et al. [50]. *Gallus gallus*, *Meleagris gallopavo*, *Taeniopygia guttata* and *Columba livia* protein sequences were downloaded from the UniProtKB database. InterProScan 5.14 was additionally used to assign gene ontologies to all annotations.

### Mitochondrial genome assembly, annotation and phylogenetic analyses

We used baiting and iterative mapping in MITObim 1.6 [54] to create an initial draft of the mitochondrial genome, using a *F. mexicanus* COI (cytochrome c oxidase subunit I) barcode sequence (AY666553) to initiate assembly. As a quality control measure, we identified mitochondrial sequence reads by blasting to the peregrine falcon mitochondrial genome (AF090338) and subsequently assembled these reads *de novo* into 38 high-quality contigs using Sequencer 5.4.6. These high-quality contigs were aligned to the MITObim assembly using Sequencer and any disagreements were resolved by eye. The final mitochondrial genome sequence was annotated using MITOS [55].

To generate a phylogenetic tree we used our *F. mexicanus* mitochondrial genome assembly and all *Falco* mitochondrial genome sequences available from NCBI (*F. peregrinus*, AF090338; *F. rusticolus*, KT989235; *F. cherrug*, KP337902; merlin, *F. columbarius*, KM264304; American kestrel, *F. sparverius*, DQ780880; common kestrel, *F. tinnunculus*, EU196361; lesser kestrel, *F. naumanni*, KM251414) and an outgroup (striated caracara, *Phalcoboenus australis*, KP064202). The latter species was chosen as an outgroup because it was the most complete and closely related mitochondrial genome available that was not of the genus *Falco*. We used CLUSTALW implemented by MEGA 7.0.21 [56] to align sequences. This alignment was used to produce a maximum likelihood tree using the GTR + G model of evolution and 1000 bootstraps.

### Phylogenetic analysis of orthologous genes

We additionally generated a phylogenetic tree using protein sequences from the three available falcon genomes (*F. cherrug*, *F. peregrinus* [57]; *F. mexicanus*, this study). For context, we additionally included sequences from all avian species available through Ensembl (*Gallus gallus*, *Meleagris gallopavo*, *Anas platyrhynchos*, *Ficedula albicollis* and *Taeniopygia guttata* [58]) as well as an outgroup (*Anolis carolinensis* [58]). Orthologous gene families were identified using BLAST® 2.3.0 and OrthoMCL 2.0.9 [59, 60]. Single-copy orthologs present in all species were extracted using custom bash scripts and aligned using MUSCLE 3.8.31 [61]. We subsequently trimmed the alignment using trimAl [62] and generated a super matrix with FASConCAT [63]. We used RAxML [64] to generate a maximum likelihood tree using the JTT + I + G + F model of evolution and 1000 bootstraps.

### SNP genotyping

We aligned the PE sequence reads back to the draft prairie falcon genome assembly using BWA [65]. We then used Picard (http://broadinstitute.github.io/picard) to sort mapped reads and identify duplicates. We used GATK 3.2 [43, 44] to identify and realign reads around insertions/deletions (indels) and subsequently call high-quality SNPs (Phred quality score ≥ 30, no more than two alleles for nuclear SNPs and a minimum depth of 10 reads) while masking indels.

We used SnpEff [66] to identify nuclear SNPs present in exonic regions, as well as predict the effects of variants on genes (i.e., amino acid changes). SNPs present in the exons of genes were annotated using BLAST® 2.2.3. We used IGV 2.3 [67, 68] to identify target SNPs with at least 60 nucleotides of high-quality flanking sequence upstream and downstream, GC content less than 65%, and no other variable sites within 20 nucleotides. We deliberately minimized linkage disequilibrium by choosing a single SNP from each annotated gene. Ultimately, we developed 190 autosomal nuclear markers from protein-coding genes. Half (95) of the gene-associated markers were specifically targeted because of evidence for selection in other species (Additional file 1: Table S1). For the remaining 95 gene-associated markers, we preferentially chose SNPs with nonsynonymous amino acid changes to increase the likelihood of identifying genes under selection, as such genes can be early indicators of population differentiation [69–72]. We additionally identified two molecular sexing markers, each of which represents a single nucleotide difference between the Z- and W-chromosomes of the CHD1 gene. All 192 markers were incorporated into a Fluidigm® SNP Type™ assay.

**Fig. 1:**
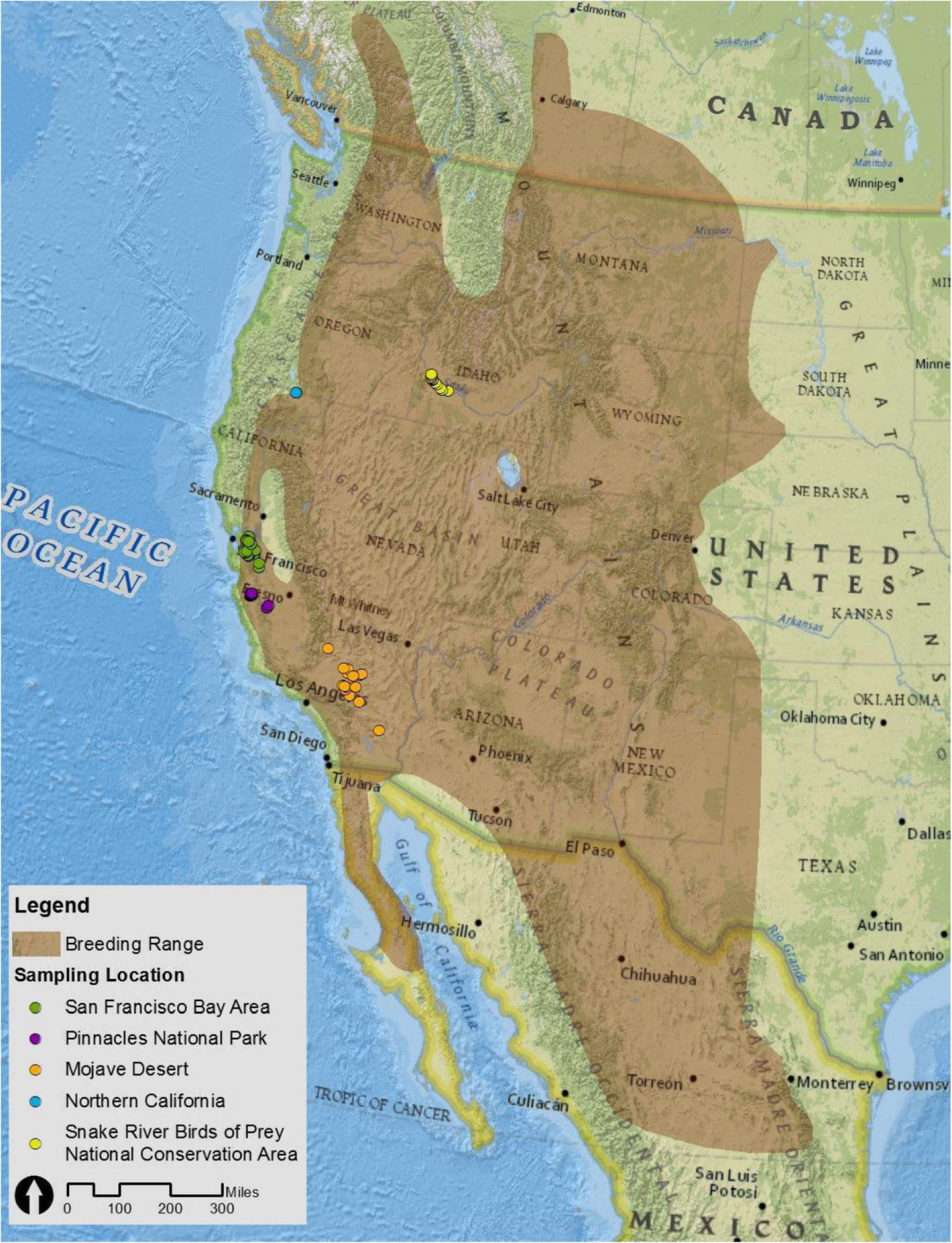
Sampling locations from San Francisco East Bay Area (CA), Pinnacles National Park (CA), the Mojave Desert (CA), Northern California and Snake River Birds of Prey National Conservation Area (ID). The map layer came from National Geographic, the breeding range layer from Birds of North America Online (https://birdsna.org), maintained by the Cornell Lab of Ornithology [20]

We genotyped 103 individual prairie falcons using the Fluidigm® BioMark HD™ Genotyping System. Blood samples were taken from 89 individuals in California and preserved in Longmire’s lysis buffer [73]. Blood samples were opportunistically collected from 14 individuals in Snake River Birds of Prey National Conservation Area in Idaho during a study of long-range movements [74]. Following sample collection, each individual prairie falcon was released. Of the 89 California individuals, 37 were sampled in and immediately around the San Francisco East Bay Area, 32 from Pinnacles National Park, 17 in the Mojave Desert and three from Northern California (Fig.1). Individuals sampled in both California and Idaho included chicks, juveniles and adults (Table 2). DNA extraction was performed using ammonium acetate [75] and potassium acetate extraction [40].

**Table 2:** Number of samples and observed and expected heterozygosities for prairie falcons sampled in Idaho and California’s San Francisco East Bay Area (East Bay), Pinnacles National Park (Pinnacles) and the Mojave Desert

	Individual sample size	Age	Sample type	Females	Males	H_O_	H_E_
California	89	Chicks, adults	Blood	40	49	0.34 ± 0.01	0.34 ± 0.01
Northern CA^a^	3	Chicks	Blood	0	3		
East Bay	37	Chicks, adults	Blood	17	20	0.33 ± 0.01	0.32 ± 0.01
Pinnacles	32	Chicks, adults	Blood	16	16	0.33 ± 0.01	0.33 ± 0.01
Mojave Desert	17	Chicks	Blood	7	10	0.34 ± 0.02	0.33 ± 0.01
Idaho	14	Juveniles, adults	Blood	13	1	0.35 ± 0.02	0.34 ± 0.01

^a^Observed and expected heterozygosity were not calculated for the three individuals from Northern California

To assess the repeatability of the assay, two additional replicates from nine individuals were also included, for a total of 121 *F. mexicanus* samples. We subsequently edited individual SNP calls using the Fluidigm® Genotyping Analysis Software. Markers were excluded from downstream analyses if: 1) data did not cluster into distinct homozygous and heterozygous states, 2) minor allele frequencies were less than 0.025 or 3) there was evidence of linkage disequilibrium (i.e., D’ > 0.20) associated with two markers, in which case only one of the two markers was removed. We calculated allele frequencies and linkage disequilibrium using the programs GenAlEx 6.501 [76] and snpStats [77].

Following Doyle et al. [5], we quantified error rates associated with SNP genotyping using three replicate samples from 9 individuals (27 samples in total). We used GenAlEx 6.501 to estimate the probability of identity (P_ID_). P_ID_ quantifies the probability that two randomly chosen individuals in a population will have identical genotypes [78] and thus indicates whether a genotyping assay can be used to assign opportunistically collected samples (e.g., feathers) to individuals. To test the accuracy of our molecular sexing approach, we determined the sex of a subset of 67 individuals using our novel markers (hereafter referred to as CHD1_1 and CHD1_2) as well as a traditional PCR (polymerase chain reaction)/gel method [79].

### Genetic variation and population structure

GenAlEx 6.501 [76] was used to calculate observed and expected heterozygosity (H_O_ and H_E_) as well as determine which loci were out of Hardy-Weinberg Equilibrium and exhibited heterozygote excess and deficiency. We tested the null hypothesis that the prairie falcons sampled are part of a single panmictic population using a combination of approaches. First, we conducted a Bayesian analysis with STRUCTURE 2.3.4 [80] and Structure Harvester [81]. Included in the analysis were 54 chicks sampled in California (i.e., individuals that have not yet had the opportunity to disperse and as such represent known-provenance birds). We subsequently conducted an additional test of panmixia using STRUCTURE 2.3.4 and 90 genotypes from both chicks and adults sampled in California and Idaho. This represents a less conservative approach (as adults may have dispersed prior to sampling) but allows us to consider population structure across a larger portion of the prairie falcon range. In both analyses, we retained only one family member genotype whenever family members were known (i.e., parent and chick or siblings) to prevent clustering algorithms from confusing family groups for population structure [82]. The 20 loci not in Hardy-Weinberg equilibrium were excluded. We considered values of K = 1–8, running each value 10 times with an initial burn-in of 100,000 MCMC (Markov chain Monte Carlo iterations) and 1,000,000 subsequent iterations for each value. We assumed an admixture ancestry model and allowed for correlated allele frequencies [83]. The results of both analyses were interpreted using mean likelihood values of K and ΔK [84]. Second, we calculated locus-specific and global pairwise F_ST_ (fixation index) values for individuals sampled in the geographically distinct regions of the San Francisco East Bay Area, Pinnacles National Park, the Mojave Desert and Snake River Birds of Prey National Conservation Area using diveRsity [85].

We used two approaches to investigate whether locus-specific signatures of natural selection were present. LOSITAN [86] was run with 500,000 replicates assuming an infinite alleles mutation model. We tested for outliers assuming a confidence interval of 0.99 and a false discovery rate (FDR) rate of 0.05. BAYESCAN [87] was initialized with 10 pilot runs of 5000 iterations and an additional burn-in of 50,000 iterations. We subsequently used a total number of 150,000 iterations (samples size of 5000 with a thinning factor of 20) to identify outlier loci by F_ST_ amongst the geographically distinct regions of the San Francisco East Bay Area, Pinnacles National Park, the Mojave Desert and Snake River Birds of Prey National Conservation Area.

## Results

### Mitochondrial and nuclear genome assembly and annotation

We generated 127 Gb of raw sequence data from *F. mexicanus*, including 41.1 Gb from the PE library, 35.0 Gb from the MP library and 50.9 Gb from the LR library (Table 1). LR fragments were assembled to form 384 LR reads. Our draft nuclear genome assembly includes 4660 scaffolds greater than 2000 bp (Table 3). These scaffolds had an N50 of 3713 kb and the longest scaffold was 17,400 kb in length. CEGMA indicated that 89% of core eukaryotic proteins were present in the draft assembly.

**Table 3:** Summary statistics for high-quality avian nuclear genomes

Species	Reference	Estimated # genes	Mean gene length	Mean exons per gene	Mean exon length	Mean intron length
*Anas platyrhynchos*	[118]	19,144	20,574	8.2	164	2664
*Coereba flaveola*	[119]	16,484	20,910	–	145	1854
*Columbia livia*	[91]	17,300	18,364	8.5	166	2271
*Falco mexicanus*	This study	16,320	16,289	9.9	148	2470
*Falco peregrinus*	[57]	16,263	20,646	8.9	173	2395
*Falco cherrug*	[57]	16,204	19,314	8.8	173	2250
*Gallus gallus*	[120]	17,040	16,702	8.0	166	2203
*Pseudopodoces humilis*	[121]	17,520	19,840	9.3	170	2208

We annotated 2181 scaffolds greater than 10 kb (N50: 3718), as shorter scaffolds rarely produce high-quality gene annotations and greatly increase computation time (C. Holt, personal communication). The PE coverage of these 2181 scaffolds (which is most relevant because only PE reads were subsequently used for SNP discovery, see below) was approximately 31X (Additional file 2: Figure S1). This process produced 16,320 gene annotations (Table 3). Mean gene length was 16,289 and on average, 9.9 exons were predicted in each gene. Mean exon and intron lengths were 148 and 2470 bp, respectively. Gene ontologies were assigned to 89% of the *F. mexicanus* genes and the top 100 protein domains can be found in Additional file 3: Table S2.

The *F. mexicanus* mitochondrial genome assembly was 17,117 bp in length and characterized by 13 protein-coding genes, two ribosomal subunit genes, 22 transfer RNA genes and a control region (Additional file 4: Figure S2). The assembled mitochondrial genome was approximately 1000 bp shorter than that of *F. peregrinus* and the hierofalcons, which can be largely attributed to a shorter pseudo-control region in *F. mexicanus*. As in many falcon species, the prairie falcon pseudo-control region was largely dominated by a repetitive region [36, 88]. As such, *F. mexicanus* may truly have a shorter pseudo-control region, as do the kestrels (e.g., *F. tinnunculus* and *F. naumanni*), or a longer repetitive region may have been collapsed during assembly. The assembly was ~ 94% identical to that of the *F. rusticulus*, *F. peregrinus* and *F. cherrug* mitochondrial genome sequences.

### Mitochondrial and nuclear phylogenetic analyses

**Fig. 2:**
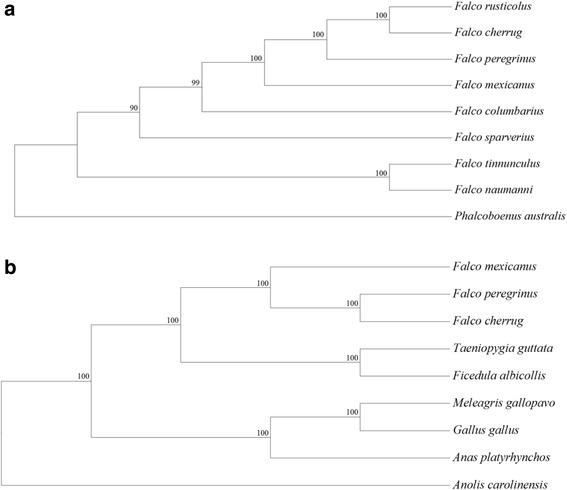
**a** A phylogeny of falcon species and an outgroup (*P. australis*) built using complete mtDNA genome sequences. A CLUSTALW alignment was used to produce a maximum likelihood tree with the GTR + G model of evolution and 1000 bootstraps. Bootstrap values < 50% are not shown on the tree. **b** A phylogeny of *F. peregrinus*, *F. cherrug*, *F. mexicanus*, *G. gallus*, *M. gallopavo*, *A. platyrhynchos*, *F. albicollis* and *T. guttata* and an outgroup (*A. carolinensis*) built using 3770 single-copy orthologs. A MUSCLE alignment was used to produce a maximum likelihood tree with the JFF + I + G + F model of evolution and 1000 bootstraps

Our maximum likelihood phylogenetic tree generated from complete mitochondrial genome sequences indicates that *F. mexicanus* is an outgroup to the clade that includes *F. peregrinus* and the hierofalcons (i.e., *F. rusticulus* and *F. cherrug*), with 100% bootstrap support for the relevant branching patterns (Fig. 2a)*.* Our OrthoMCL analysis identified 3770 single-copy orthologs present in all 9 species. Broader phylogenetics relationships among avian species echoed those of recent publications (e.g., the chicken, turkey and duck form an evolutionary branch distinct from that of the falcons, zebra finch and collared flycatcher [57, 89, 90]; Fig. 2b). The maximum likelihood phylogenetic tree generated from nuclear protein-coding sequences again indicates that *F. mexicanus* is an outgroup to the clade that includes *F. peregrinus* and the hierofalcon *F. cherrug* (Fig. 2b).

### SNP assay development and genotyping

We initially identified 567,599 high-quality SNPs. Of these, 7401 were found in the exons of genes. As described in the methods, the 190 autosomal nuclear markers subsequently included in our SNP assay were chosen for their high-quality flanking sequence, to minimize linkage disequilibrium and maximize the likelihood of identifying genes under selection. Following genotyping, we excluded from downstream analysis 47 loci for reasons outlined in the methods (e.g., minor allele frequencies less than 0.025). Of the remaining 143 loci used to generate all results described below, at least 133 loci amplified for each of the 103 prairie falcons genotyped.

Our error rate, calculated following Doyle et al. [5] and based on three replicate samples taken from each of 9 individuals, was 0.3%. P_ID_ was estimated as 1.1 × 10^− 43^. Our CHD1_1 and CHD1_2 sexing markers were 92 and 100% concordant with Fridolfsson and Ellegren’s [79] PCR/gel molecular sexing method, respectively. All instances of disagreement between CHD1_1 and other molecular sexing methods indicated allelic dropout (i.e., females misidentified as males). CHD1_2 was therefore used for all subsequent molecular sexing. Of the 103 prairie falcons genotyped, 53 were female and 50 male (Table 2).

### Heterozygosity and population structure

Mean H_O_ and H_E_ at autosomal SNPs were both 0.34 ± 0.01 SE. Of the 143 nuclear loci considered, 20 were out of Hardy-Weinberg Equilibrium. F_IS_ (inbreeding coefficient) values for these 20 SNPs ranged from − 0.31 to 0.50, with 14 markers showing evidence of heterozygote deficiency and 6 showing evidence of heterozygote excess. When samples from California and Idaho are considered separately, average H_O_ and H_E_ varied from 0.33 ± 0.01 SE to 0.35 ± 0.02 SE and 0.32 ± 0.01 SE to 0.34 ± 0.01 SE, respectively (Table 2).

**Fig. 3:**
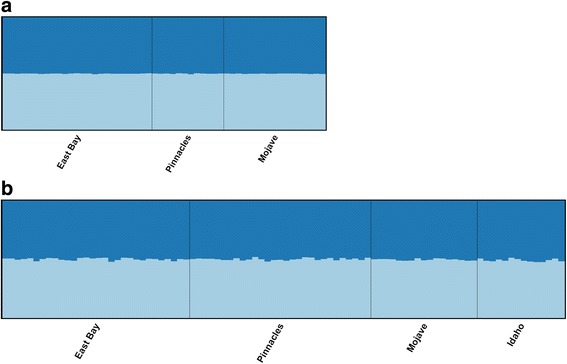
STRUCTURE results consistent with panmixia (i.e., K=1) for known and unknown-provenance falcons. **a** Results of STRUCTURE analysis for 54 known-provenance chicks sampled from California’s San Francisco East Bay Area, Pinnacles National Park and the Mojave Desert that were genotyped at 123 SNP loci. STRUCTURE results were CLUMPP-averaged across 10 runs when K is assumed to be equal to two. **b** Results of STRUCTURE analysis for a mix of 90 known and unknown provenance individuals sampled in California and Idaho and genotyped at 123 SNP loci. STRUCTURE results were CLUMPP-averaged across 10 runs when K is assumed to be equal to two

Both STRUCTURE analyses (i.e., conservative and relaxed approaches) provide evidence that individual prairie falcons in California and Idaho make up a single, panmictic population (Fig. 3a and b, Additional file 5: Figure S3). Mean likelihood values of K are greatest for K = 1 in both instances. Global pairwise F_ST_ values for four putative populations (i.e., the San Francisco East Bay Area, Pinnacles National Park, the Mojave Desert and Snake River Birds of Prey National Conservation Area) ranged from 0.01 to 0.03 and did not indicate significant genetic differentiation (Table 4). Our LOSITAN analysis identified two outlier SNPs potentially under directional selection and associated with genes CACNA1G and A2ML1 (Additional file 6:Figure S4). BAYESCAN did not detect any statistically significant outlier loci, however the SNP associated with A2ML1 showed clear differentiation from other markers (Additional file 6: Figure S4). Locus-specific pairwise F_ST_ comparisons for A2ML1 indicate high levels of genetic differentiation (i.e., F_ST_ > 0.10) between the San Francisco East Bay Area and Idaho, the San Francisco East Bay Area and the Mojave Desert, Idaho and Pinnacles National Park and the Mojave Desert and Pinnacles National Park (Additional file 7: Table S3).

**Table 4:** Mean F_ST_ values and 95% CI for each pairwise comparison

Pairwise comparison	Global FST	95% CI
East Bay vs. Idaho^a^	0.03	0.01–0.05
East Bay vs. Mojave	0.02	0–0.03
East Bay vs. Pinnacles	0.01	0–0.02
Idaho vs. Mojave	0.01	−0.02 – 0.03
Idaho vs. Pinnacles	0.02	0.01–0.05
Mojave vs. Pinnacles	0.01	0–0.03

^a^Sampling sites include Idaho and California’s San Francisco East Bay Area (East Bay), Pinnacles National Park (Pinnacles) and the Mojave Desert (Mojave)

## Discussion

### Nuclear and mitochondrial genome assembly, annotation and phylogenetics

Herein, we describe the draft genome assembly of *F. mexicanus*, a species for which population-level genetic variability is undocumented and phylogenetic relationships to other falcons contested. The assembly size (1.17 Gb) and the number of genes annotated (> 16,000) are very similar to that of the *F. peregrinus* and *F. cherrug* genomes ([57]; Table 3). The overall completeness of the genome is further indicated by the number of core eukaryotic genes identified (89%), which is comparable to other high quality avian genome assemblies (e.g., rock pigeon [91]).

Our maximum likelihood phylogenetic trees utilizing complete mitochondrial genome sequences and nuclear protein-coding sequences position *F. mexicanus* as an outgroup to the clade that includes *F. peregrinus* and the hierofalcons (as in [39]) rather than as a sister species to *F. peregrinus* (as in [36, 37]). As such, the ecological and morphological similarities between *F. mexicanus* and the hierofalcons (e.g., syringeal characters [92]) might simply be conserved characters present in many falcon species, rather than evidence of a close evolutionary relationship. It should be reiterated, however, that in our nuclear phylogeny the hierofalcons are represented by a single species (*F. cherrug*) and additional sequencing will pave the way for fine-scale resolution of branching patterns within Falconinae as well as Neoaves as a whole. For example, an orthologous gene set of protein-coding genes, introns and nonoverlapping ultraconserved elements illustrated that falcons, although traditionally grouped with other diurnal raptors, are more closely related to seriemas, parrots and members of Passeriformes ([90], see also [89]). More accurate estimates of branch lengths, in turn, can improve our estimates of evolutionary distinctiveness, allowing conservation priority to be assigned to species based not just on conservation status (e.g., IUCN rankings) but also by how much evolutionary information would be lost if the species became extinct.

### SNP assay development and genotyping

Common molecular approaches (e.g., genotyping with a species-specific suite of microsatellite markers) have been underutilized in *F. mexicanus*. As a result, little is known about the population genetics of the species throughout its range. Our novel SNP assay is a powerful tool in addressing gaps in our understanding. As with assays designed for golden eagles [5] and grey whales [93], SNP genotyping produced both a low error rate and P_ID_ (probability of identity). A low P_ID_ indicates that, for example, two naturally shed feathers with identical genotypes were likely derived from the same individual and could be so assigned. As a result, our approach can be applied to noninvasive sampling in addition to the genotyping of high-quality samples taken from known individuals (as practiced in this study). Noninvasive sampling and subsequent DNA extraction from naturally shed hair, feathers, fecal matter and carcasses has facilitated studies of dispersal (wolves, *Canis lupis* [94]), population size (brown bears, *Ursus arctos* [95]), sex ratio (Eurasian otter, *Lutra lutra* [96]), movement (white-tailed eagles, *Haliaeetus albicilla* [97]), mating systems, population turnover and behavior (imperial eagles, *Aquila heliaca* [76, 98]).

Additionally, our assay incorporates a molecular sexing marker that is in complete accordance with traditional molecular sexing methods. Finally, the incorporation of ~ 140 gene-associated SNPs has a number of potential benefits. For example, heterozygosity estimated from a large suite of SNPs may reflect genome-wide genetic variation more accurately than other methods (e.g., microsatellites [99]), facilitating future studies of heterozygosity-fitness correlations.

### Genetic variation and population structure

We tested the null hypothesis that prairie falcons in the western United States make up a single, interbreeding population, as well as the alternative hypothesis that genetically distinct populations exist. There are biological arguments for each scenario. Most avian species are highly mobile, capable of long-distance movement and able to surmount landscape features that act as barriers to other species (e.g., mountain ranges, rivers), promoting gene flow. As a result, species such as mallards (*Anas platyrhynchos*) and turtle doves (*Streptopelia turtur*) exhibit little to no population structure even at a continental level [100, 101]. However, mobility does not necessarily indicate dispersal to and inclusion in novel breeding populations. Avian species can also exhibit natal philopatry and site fidelity that interrupts gene flow and contributes to population structure (e.g., black-browed albatrosses, *Thalassarche melanophris* [102]; saltmarsh sparrows, *Ammodramus caudacutus* [103]; penguins, *Pygoscelis papua* [104]; white-tailed sea eagles, *Haliaeetus albicilla* [105]).

Banding and telemetry data gives us an indication of *F. mexicanus* mobility and dispersal. Prairie falcons breeding in Canada and Idaho are known to migrate up to 1900 and 4600 km (kilometers), respectively [74, 106, 107], indicating an ability to travel long distances. However, nestlings banded at Snake River Birds of Prey National Conservation Area have a relatively conservative mean dispersal distance from natal to breeding territories of ~ 9 km [108]. Adult prairie falcons also show a tendency toward breeding territory fidelity. For example, telemetry data indicates that *most* adult prairie falcons studied at Snake River Birds of Prey National Conservation Area are loyal to their nesting sites across years (i.e., return to within 2.5 km of the previous year’s nesting site; Steenhof et al. [74]). However, exceptions occur. For example, Steenhof et al. [74] documented one of 24 telemetered prairie falcons dispersing between breeding locations 124 km from one another across two years. Relatively few dispersing individuals are required to genetically homogenize populations [109, 110], so even this low level of long-distance movement between breeding locations may be enough to result in a genetically panmictic population. This likely explains the lack of population structure we see throughout California. Additional sampling, however, will be required to determine whether the lack of structure we see between California and Idaho is indicative of the entire western prairie falcon range.

Despite specifically targeting loci likely to be under selection and identifying 20 loci with departures from Hardy-Weinberg equilibrium, our LOSITAN and BAYESCAN analyses identified just two potential outlier loci (CACNA1G and A2ML1) following FDR correction for multiple testing. We will focus the remainder of our discussion on the SNP associated with A2ML1, given the relatively consistent signals of selection from both LOSITAN and BAYESCAN analyses. For this SNP, pairwise F_ST_ values indicate that individuals sampled in the San Francisco East Bay Area and Pinnacles National Park differ genetically from individuals sampled in the Mojave Desert and Idaho. A2ML1 is a gene that encodes for a protein that inhibits proteases and is associated with successful embryonic development in chickens and ducks [111, 112]. Interestingly, A2ML1 is considered a candidate reproductive barrier gene isolating the Italian sparrow (*Passer italiae*) from its two progenitor species: the house and Spanish sparrows (*Passer domesticus* and *Passer hispaniolensis*, respectively). Allele frequencies associated with A2ML1 exhibit steep clines throughout the range of the three sparrow species [113, 114]. Although the majority of our analyses indicate that prairie falcons might be managed as a single population, it is possible that the segregating allele frequencies associated with A2ML1 are an early signal of population divergence, as studies have shown that loci under selection show more structure between populations than neutral loci [70]. However, given our small sample size, additional sampling will be required to confirm these results. Furthermore, incorporating markers with different mutation rates and effective population sizes (e.g., intergenic SNPs, microsatellites, mitochondrial sequences) will further expand our understanding of genetic differentiation in the prairie falcon.

## Conclusions

Our study illustrates how genomic resources can rapidly shed light on genetic variability at the species- and population-level in understudied species. Our evidence that the prairie falcon is neither sister taxon to the peregrine falcon nor member of the hierofalcons illustrates how a genomic tool set can resolve phylogenies, ultimately contributing to more accurate estimates of evolutionary distinctiveness. Furthermore, our preliminary results largely demonstrate panmixia in the prairie falcon and imply that management actions undertaken to benefit the species at the local level (e.g., regional or park level) have the potential to influence the species as a whole. For example, panmixia indicates a putative tendency for *F. mexicanus* to disperse throughout its range. This may serve to recover populations locally extirpated as a result of development [26], similar to the sources-sink dynamics demonstrated for recovering peregrine falcon populations in California [115, 116] or the recolonization of volcanic islands post-eruption [117]. Lastly, our sequencing of the prairie falcon genome provides the raw data for subsequent studies of repetitive elements, chromosomal organization and many other research avenues.

## Additional files

Additional file 1: **Table S1.** Description of 96 prairie falcon SNPs associated with genes under selection in different species. (PDF 299 kb)

Additional file 2: **Figure S1.** Paired-end read coverage of 2181 scaffolds. Sequencing depth is on the x-axis while the y-axis shows the percentage of total bases at a given depth. Reads were aligned to the genome using BWA. (PDF 156 kb)

Additional file 3: **Table S2.** Top Pfam domain hits in the *F. mexicanus* genome and their counts. (PDF 175 kb)

Additional file 4: **Figure S2.** The *F. mexicanus* mitochondrial genome map. COX1, COX2 and COX3 indicate cytochrome oxidase subunits 1–3; CYTB indicates cytochrome b; atp6 and atp8 indicate ATPase subunits 6 and 8; ND1–ND6 indicate NADH dehydrogenase subunits 1–6. Transfer RNA genes are designated by single-letter amino acid codes. (PDF 192 kb)

Additional file 5: **Figure S3.** Mean estimated Ln probability of data ± SD for K 1 through 8, averaged across 10 runs, for known and unknown-provenance falcons. A) Results of STRUCTURE analysis for 54 known-provenance chicks sampled from California’s San Francisco East Bay Area, Pinnacles National Park and the Mojave Desert that were genotyped at 123 SNP loci. B) Results of STRUCTURE analysis for a mix of 90 known and unknown provenance individuals sampled in California and Idaho and genotyped at 123 SNP loci. (PDF 268 kb)

Additional file 6: **Figure S4.** Results of LOSITAN and BAYESCAN analyses. a) The confidence area for candidate loci under positive selection is shown in red. LOSITAN identified two outlier loci: X1613239_349604 (CACNA1G) and X1613580_160905 (A2ML1). b) No outlier loci were detected via BAYESCAN analysis. Posterior odds are plotted on the x axis and F_ST_ index values on the y axis. (PDF 36 kb)

Additional file 7: **Table S3.** Locus-specific pairwise F_ST_ values for prairie falcons in the San Francisco East Bay Area (East Bay), Pinnacles National Park (Pinnacles), the Mojave Desert (Mojave) and Idaho. (PDF 281 kb)

## Abbreviations

bp: Basepairs
COI: Cytochrome c oxidase subunit I
DNA: Deoxyribonucleic acid
EST: Expressed sequence tag
FDR: False discovery rate
F_IS_: Inbreeding coefficient
F_ST_: Fixation index
Gb: Gigabases
H_E_: Expected heterozygosity
H_O_: Observed heterozygosity
kb: Kilobases
km: Kilometers
LR: Long reads
MCMC: Markov chain Monte Carlo iterations
MP: Mate-paired
PCR: Polymerase chain reaction
PE: Paired-end
P_ID_: Probability of identity
SNP: Single nucleotide polymorphism
