# Supplementary material for: New insights into the phylogenetics and population structure of the prairie falcon (Falco mexicanus)
Source: BMC Genomics. 2018 Apr 4;19:233. doi: 10.1186/s12864-018-4615-z (PMC5885362; doi:10.1186/s12864-018-4615-z)
Supplement: Supplementary file 1 — Table S1. Description of 96 prairie falcon SNPs associated with genes under selection in different species. (PDF 299 kb) [file 12864_2018_4615_MOESM1_ESM.pdf]

Additional file 1: Supplementary Table 1. Description of 96 prairie falcon SNPs associated with genes under selection in different species.

SNP ID	Ontology	Organism	Citation
1614105:566001	ABHD6	*Alligator sinensis*	[1]
1614686:5293396	ACADS	*Alligator sinensis*	[1]
1612666:3119752	ADAM19	*Alligator sinensis*	[1]
1614105:5966489	ADAMTS9	*Alligator sinensis*	[1]
1615281:1321851	AGA	*Taeniopygia guttata*	[2]
1612891:582304	AIM1	*Alligator sinensis*	[1]
1612562:4811621	AIMP2	*Alligator sinensis*	[1]
1613640:779706	ANO10	*Geospiza magnirostris*	[3]
1615259:181134	AQP9	*Pseudopodoces humilis*	[4]
1614105:247653	ASB14	*Chrysemys picta*	[5]
1612964:1419399	ASPN	*Alligator sinensis*	[1]
1612469:1301514	ATXN1	*Taeniopygia guttata*	[2]
1612469:1302424	ATXN1	*Taeniopygia guttata*	[2]
1612403:887669	BICD2	*Taeniopygia guttata*	[6]
1613897:10629663	C8B	*Alligator sinensis*	[1]
1614075:3255035	CACNA1B	*Taeniopygia guttata*	[6]
1613384:1458567	CACNA1D	*Taeniopygia guttata*	[2]
1614520:2614106	CACNA1G	*Taeniopygia guttata*	[6]
1612694:6471166	CHD2	*Pseudopodoces humilis*	[4]
1612844:1330017	CLASP2	*Taeniopygia guttata*	[6]
1612520:6017799	CNTN1	*Alligator sinensis*	[1]
1613106:1290319	CTSE	*Alligator sinensis*	[1]
1612096:1127622	DAGLA	*Alligator sinensis*	[1]
1613785:129109	DNAJC6	*Taeniopygia guttata*	[6]
1614282:1141302	EML4	*Gallus gallus*	[7]
1612929:63221	EPCAM	*Alligator sinensis*	[1]
1614131:6223675	ERCC5	*Taeniopygia guttata*	[6]
1612439:222666	EZH2	*Pseudopodoces humilis*	[4]
1612346:15212015	F10	*Alligator sinensis*	[1]
1615288:3118567	FLII	*Alligator sinensis*	[1]
1612932:487927	GRIA2	*Taeniopygia guttata*	[2]
1613580:5715670	GSK3B	*Falco peregrinus*	[8]
1612617:568182	HADHA	*Alligator sinensis*	[1]
1613005:949397	HIF1AN	*Parus humilis*	[9]
1612289:10157266	HPS6	*Alligator sinensis*	[1]
1593808:8041	IGF2R	*Geospiza magnirostris*	[3]
1614688:1973115	IL1RAP	*Alligator sinensis*	[1]
1612289:1336404	INPP5F	*Pseudopodoces humilis*	[4]
1612432:234418	ITGA9	*Alligator sinensis*	[1]
1611951:4700267	KDR	*Alligator sinensis*	[1]
1614506:145763	LARP7	*Alligator sinensis*	[1]

1613897:9109052	LRP8	*Alligator sinensis*	[1]
1614314:3008240	LRRC16A	*Taeniopygia guttata*	[6]
1612503:840990	LRRC7	*Parus humilis*	[9]
1614643:1891609	LRRC8D	*Alligator sinensis*	[1]
1612694:9639101	LRRK1	*Parus humilis*	[9]
1612666:6324471	MAPK9	*Falco peregrinus*	[8]
1612346:11450432	MFSD9	*Alligator sinensis*	[1]
1611802:3456222	MPHOSPH10	*Alligator sinensis*	[1]
1612632:1214371	MYH10	*Taeniopygia guttata*	[6]
1613669:4872333	NID1	*Alligator sinensis*	[1]
1613408:287251	NLGN3	*Alligator sinensis*	[1]
1612346:10672156	NPAS2	*Taeniopygia guttata*	[2]
1613274:21823	OPN3	*Alligator sinensis*	[1]
1615277:1808636	PAPSS2	*Alligator sinensis*	[1]
1612441:3381811	PDE1A	*Taeniopygia guttata*	[6]
1613056:1192015	PIGW	*Pseudopodoces humilis*	[4]
1614298:1387066	PLA2G6	*Taeniopygia guttata*	[2]
1613518:109196	PLEKHA8	*Alligator sinensis*	[1]
1614310:2068237	PLOD2	*Gallus gallus*	[7]
1613026:97585	PLP1	*Taeniopygia guttata*	[2]
1615221:81774	POU1F1	*Geospiza magnirostris*	[3]
1611833:26797	PSMD2	*Parus humilis*	[9]
1613897:3358312	PTPRF	*Taeniopygia guttata*	[2]
1612196:132010	RBM5	*Taeniopygia guttata*	[6]
1612849:6445227	RCN1	*Alligator sinensis*	[1]
1614664:1823882	RLF	*Alligator sinensis*	[1]
1613596:4590629	ROBO2	*Gallus gallus*	[7]
1613179:594551	RPS3A	*Alligator sinensis*	[1]
1612204:4622386	SAPCD2	*Alligator sinensis*	[1]
1612849:741062	SHANK2	*Gallus gallus*	[7]
1613381:334429	SLC26A3	*Alligator sinensis*	[1]
1612041:807987	SLC4A1	*Alligator sinensis*	[1]
1613314:1496853	SMC1B	*Alligator sinensis*	[1]
1614264:2224382	SMO	*Alligator sinensis*	[1]
1614271:1810603	SMOC1	*Taeniopygia guttata*	[6]
1613751:1071374	SORBS2	*Taeniopygia guttata*	[6]
1614520:2781608	SPATA20	*Alligator sinensis*	[1]
1613464:1822371	SPG11	*Alligator sinensis*	[1]
1614474:953448	ST6GALNAC1	*Pseudopodoces humilis*	[4]
1612628:802176	SUCNR1	*Gallus gallus*	[7]
1613790:1792316	TADA2B	*Pseudopodoces humilis*	[4]
1614305:2995114	TBC1D1	*Gallus gallus*	[7]
1613666:3047902	TEX2	*Taeniopygia guttata*	[6]
1612844:2076748	TGM4	*Alligator sinensis*	[1]
1612100:10539	TLK2	*Taeniopygia guttata*	[6]

1612142:1217548	TLR5	*Alligator sinensis*	[1]
1612142:1004236	TP53BP2	*Taeniopygia guttata*	[6]
1614362:1546835	TSNARE1	*Gallus gallus*	[7]
1613325:211217	UBR1	*Taeniopygia guttata*	[6]
1614520:3065971	USH1G	*Alligator sinensis*	[1]
1612562:4406105	VWA3A	*Alligator sinensis*	[1]
1613514:1252656	WNK1	*Taeniopygia guttata*	[2]
1613314:1101621	WNT7B	*Parus humilis*	[9]
1613342:9318881	ZNFX1	*Pseudopodoces humilis*	[4]

Literature cited

1. Wan Q-H, Pan S-K, Hu L, Zhu Y, Xu P-W, Xia J-Q, Chen H, He G-Y, He J, Ni X-W, Hou H-L, Liao S-G, Yang H-Q, Chen Y, Gao S-K, Ge Y-F, Cao C-C, Li P-F, Fang L-M, Liao L, Zhang S, Wang M-Z, Dong W, Fang S-G. Genome analysis and signature discovery for diving and sensory properties of the endangered Chinese alligator. Cell Res 2013, 23:1091–105.

2. Nam K, Mugal C, Nabholz B, Schielzeth H, Wolf JBW, Backström N, Künstner A, Balakrishnan CN, Heger A, Ponting CP, Clayton DF, Ellegren H. Molecular evolution of genes in avian genomes. Genome Biol 2010, 11:R68.

3. Rands CM, Darling A, Fujita M, Kong L, Webster MT, Clabaut C, Emes RD, Heger A, Meader S, Hawkins MB, Eisen MB, Teiling C, Affourtit J, Boese B, Grant PR, Grant BR, Eisen JA, Abzhanov A, Ponting CP. Insights into the evolution of Darwin's finches from comparative analysis of the *Geospiza magnirostris* genome sequence. BMC Genomics 2013, 14:95.

4. Cai Q, Qian X, Lang Y, Luo Y, Xu J, Pan S, Hui Y, Gou C, Cai Y, Hao M, Zhao J, Wang S, Wang Z, Zhang X, He R, Liu J, Luo L, Li Y, Wang J. Genome sequence of ground tit *Pseudopodoces humilis* and its adaptation to high altitude. Genome Biol 2013, 14:R29.

5. Shaffer HB, Minx P, Warren DE, Shedlock AM, Thomson RC, Valenzuela N, Abramyan J, Amemiya CT, Badenhorst D, Biggar KK, Borchert GM, Botka CW, Bowden RM, Braun EL, Bronikowski AM, Bruneau BG, Buck LT, Capel B, Castoe T a, Czerwinski M, Delehaunty KD, Edwards S V, Fronick CC, Fujita MK, Fulton L, Graves TA, Green RE, Haerty W, Hariharan R, Hernandez O, et al. The western painted turtle genome, a model for the evolution of extreme physiological adaptations in a slowly evolving lineage. Genome Biol 2013, 14:R28.

6. Warren WC, Clayton DF, Ellegren H, Arnold AP, Hillier LW, Künstner A, Searle S, White S, Vilella AJ, Fairley S, Heger A, Kong L, Ponting CP, Jarvis ED, Mello C V, Minx P, Lovell P, Velho TAF, Ferris M, Balakrishnan CN, Sinha S, Blatti C, London SE, Li Y, Lin Y-C, George J, Sweedler J, Southey B, Gunaratne P, Watson M, et al. The genome of a songbird. Nature 2010, 464:757–62.

7. Rubin C-J, Zody MC, Eriksson J, Meadows JRS, Sherwood E, Webster MT, Jiang L, Ingman M, Sharpe T, Ka S, Hallböök F, Besnier F, Carlborg O, Bed'hom B, Tixier-Boichard M, Jensen P, Siegel P, Lindblad-Toh K, Andersson L. Whole-genome resequencing reveals loci under selection during chicken domestication. Nature 2010, 464:587–591.

8. Zhan X, Pan S, Wang J, Dixon A, He J, Muller MG, Ni P, Hu L, Liu Y, Hou H, Chen Y, Xia J, Luo Q, Xu P, Chen Y, Liao S, Cao C, Gao S, Wang Z, Yue Z, Li G, Yin Y, Fox NC, Wang J, Bruford MW. Peregrine and saker falcon genome sequences provide insights into evolution of a predatory lifestyle. Nat Genet 2013, 45:563–6.

9. Qu Y, Zhao H, Han N, Zhou G, Song G, Gao B, Tian S, Zhang J, Zhang R, Meng X, Zhang Y, Zhang Y, Zhu X, Wang W, Lambert D, Ericson PGP, Subramanian S, Yeung C, Zhu H, Jiang Z, Li R, Lei F. Ground tit genome reveals avian adaptation to living at high altitudes in the Tibetan plateau. Nat Commun 2013, 4:2071.
