# Supplementary material for: New insights into the phylogenetics and population structure of the prairie falcon (Falco mexicanus)
Source: BMC Genomics. 2018 Apr 4;19:233. doi: 10.1186/s12864-018-4615-z (PMC5885362; doi:10.1186/s12864-018-4615-z)

Additional file 2: Supplementary Figure 1. Paired-end read coverage of 2,181 scaffolds. Sequencing depth is on the x-axis while the y-axis shows the percentage of total bases at a given depth. Reads were aligned to the genome using BWA.

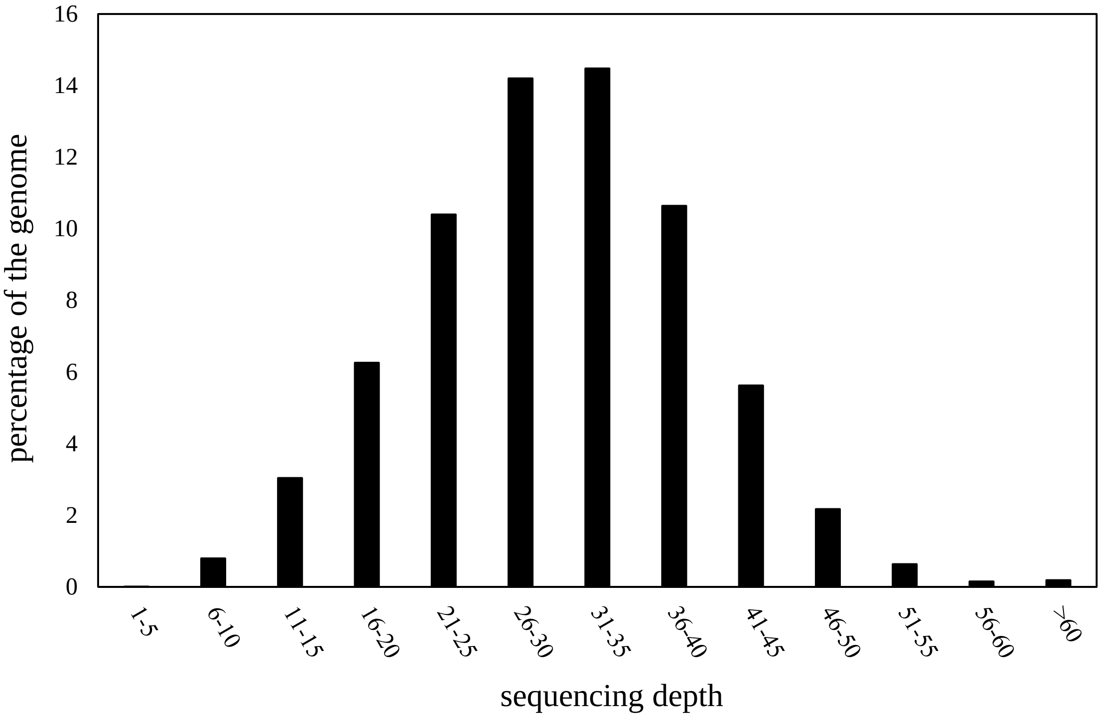

Supplement: Supplementary file 2 — Figure S1. Paired-end read coverage of 2181 scaffolds. Sequencing depth is on the x-axis while the y-axis shows the percentage of total bases at a given depth. Reads were aligned to the genome using BWA. (PDF 156 kb) [file 12864_2018_4615_MOESM2_ESM.pdf]
