# Supplementary material for: New insights into the phylogenetics and population structure of the prairie falcon (Falco mexicanus)
Source: BMC Genomics. 2018 Apr 4;19:233. doi: 10.1186/s12864-018-4615-z (PMC5885362; doi:10.1186/s12864-018-4615-z)
Supplement: Supplementary file 3 — Table S2. Top Pfam domain hits in the F. mexicanus genome and their counts. (PDF 175 kb) [file 12864_2018_4615_MOESM3_ESM.pdf]

Additional file 3: Supplementary Table 2. Top Pfam domain hits in the *F. mexicanus* genome and their counts.

**Pfam Domain**	**Count**
Immunoglobulin I-set domain	575
Fibronectin type III domain	517
Cadherin domain	476
WD domain, G-beta repeat	461
Protein kinase domain	380
Collagen triple helix repeat (20 copies)	288
7 transmembrane receptor (rhodopsin family)	285
Leucine rich repeat	281
Ankyrin repeats (3 copies)	277
RNA recognition motif.	227
Calcium-binding EGF domain	212
Homeobox domain	200
Kelch motif	197
EGF-like domain	194
Sushi repeat (SCR repeat)	193
PDZ domain (Also known as DHR or GLGF)	192
C2 domain	176
BTB/POZ domain	170
Ion transport protein	165
Thrombospondin type 1 domain	155
Tetratricopeptide repeat	155
Low-density lipoprotein receptor domain class A	150
PH domain	148
Spectrin repeat	143
Ras family	140
Immunoglobulin domain	131
Laminin EGF domain	129
CUB domain	127
LIM domain	122
Protein tyrosine kinase	117
Variant SH3 domain	110
Nebulin repeat	109
Mitochondrial carrier protein	103
Immunoglobulin V-set domain	102
EF-hand domain pair	99
C2H2-type zinc-finger domain	98
Myosin head (motor domain)	97

Ankyrin repeats (many copies)	95
IQ calmodulin-binding motif	90
SAM domain (Sterile alpha motif)	88
Laminin G domain	88
Low-density lipoprotein receptor repeat class B	87
SH3 domain	86
Armadillo/beta-catenin-like repeat	86
Helix-loop-helix DNA-binding domain	85
Zinc finger, C2H2 type	84
Trypsin	84
Zinc-finger of C2H2 type	83
SH2 domain	82
Helicase conserved C-terminal domain	82
Scavenger receptor cysteine-rich domain	80
KH domain	78
C2H2-type zinc finger	78
Zinc finger, C3HC4 type (RING finger)	77
TPR repeat	77
von Willebrand factor type A domain	76
Lectin C-type domain	74
Plectin repeat	73
RhoGAP domain	73
Regulator of chromosome condensation (RCC1) repeat	72
Calponin homology (CH) domain	72
ABC transporter	68
Coagulation Factor Xa inhibitory site	65
Intermediate filament protein	65
Major Facilitator Superfamily	59
von Willebrand factor type D domain	59
Protein-tyrosine phosphatase	59
Cytochrome P450	59
Kazal-type serine protease inhibitor domain	58
RhoGEF domain	58
BTB And C-terminal Kelch	56
short chain dehydrogenase	56
Cadherin-like	55
Calx-beta domain	53
Ubiquitin carboxyl-terminal hydrolase	53
ATPase family associated with various cellular activities	52
Filamin/ABP280 repeat	50
DEAD/DEAH box helicase	49

MORN repeat	48
WW domain	48
HMG (high mobility group) box	47
Receptor family ligand binding region	46
EF hand	46
Neurotransmitter-gated ion-channel transmembrane region	45
Kinesin motor domain	45
AMP-binding enzyme	45
SPRY domain	45
FERM central domain	45
Neurotransmitter-gated ion-channel ligand binding domain	44
AAA domain	43
Annexin	43
C8 domain	42
Bromodomain	41
7 transmembrane receptor (Secretin family)	41
PX domain	41
PHD-finger	41
Ligand-binding domain of nuclear hormone receptor	41
Olfactory receptor	41
Hemopexin	40
Thioredoxin	40
