# Supplementary material for: New insights into the phylogenetics and population structure of the prairie falcon (Falco mexicanus)
Source: BMC Genomics. 2018 Apr 4;19:233. doi: 10.1186/s12864-018-4615-z (PMC5885362; doi:10.1186/s12864-018-4615-z)
Supplement: Supplementary file 4 — Figure S2. The F. mexicanus mitochondrial genome map. COX1, COX2 and COX3 indicate cytochrome oxidase subunits 1–3; CYTB indicates cytochrome b; atp6 and atp8 indicate ATPase subunits 6 and 8; ND1–ND6 indicate NADH dehydrogenase subunits 1–6. Transfer RNA genes are designated by single-letter amino acid codes. (PDF 192 kb) [file 12864_2018_4615_MOESM4_ESM.pdf]

Additional file 4: Supplementary Figure 2. The *F. mexicanus* mitochondrial genome map. COX1, COX2 and COX3 indicate cytochrome oxidase subunits 1–3; CYTB indicates cytochrome b; ATP6 and ATP8 indicate ATPase subunits 6 and 8; ND1–ND6 indicate NADH dehydrogenase subunits 1–6.

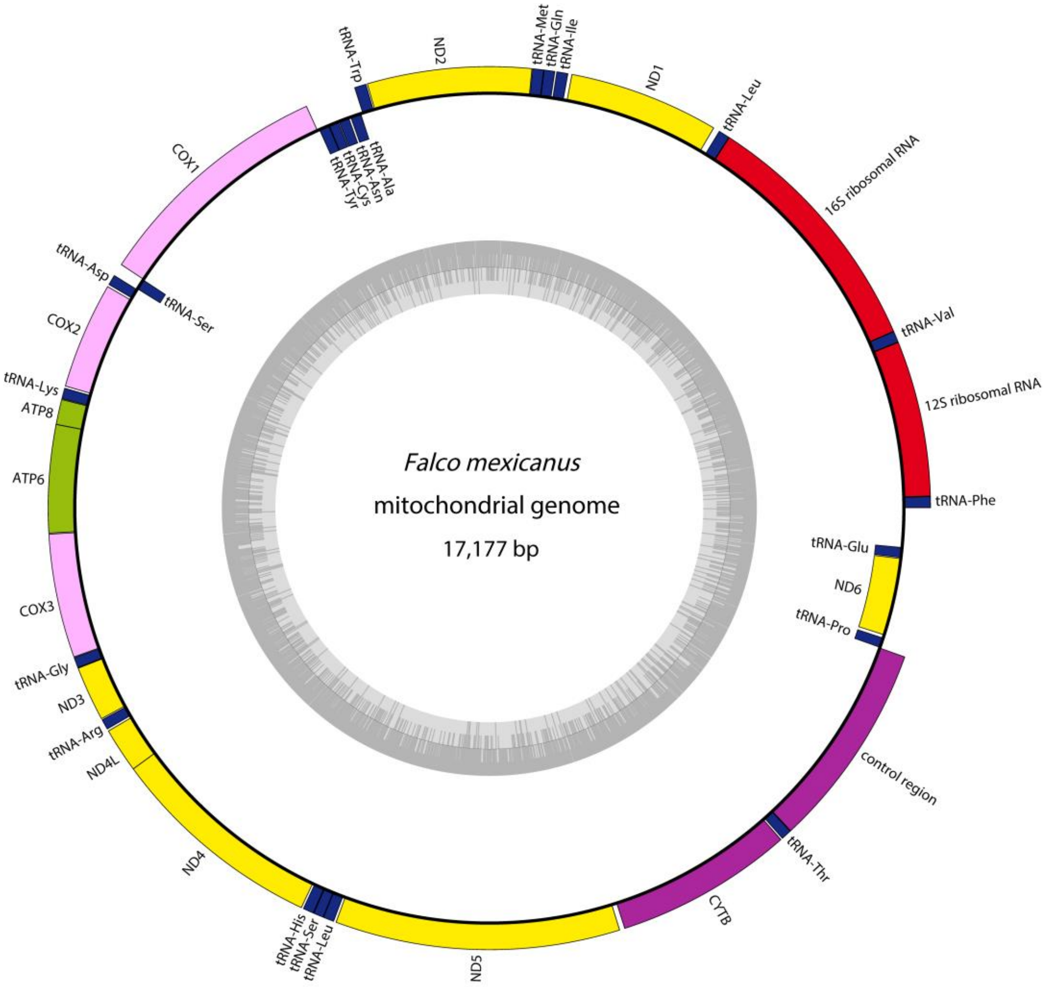

.
