# Supplementary material for: New insights into the phylogenetics and population structure of the prairie falcon (Falco mexicanus)
Source: BMC Genomics. 2018 Apr 4;19:233. doi: 10.1186/s12864-018-4615-z (PMC5885362; doi:10.1186/s12864-018-4615-z)

Additional file 5: Supplementary Figure 3. Mean estimated Ln probability of data ± SD for K 1 through 8, averaged across 10 runs, for known and unknown-provenance falcons. A) Results of STRUCTURE analysis for 54 known-provenance chicks sampled from California's San Francisco East Bay Area, Pinnacles National Park and the Mojave Desert that were genotyped at 123 SNP loci. B) Results of STRUCTURE analysis for a mix of 90 known and unknown provenance individuals sampled in California and Idaho and genotyped at 123 SNP loci.

A

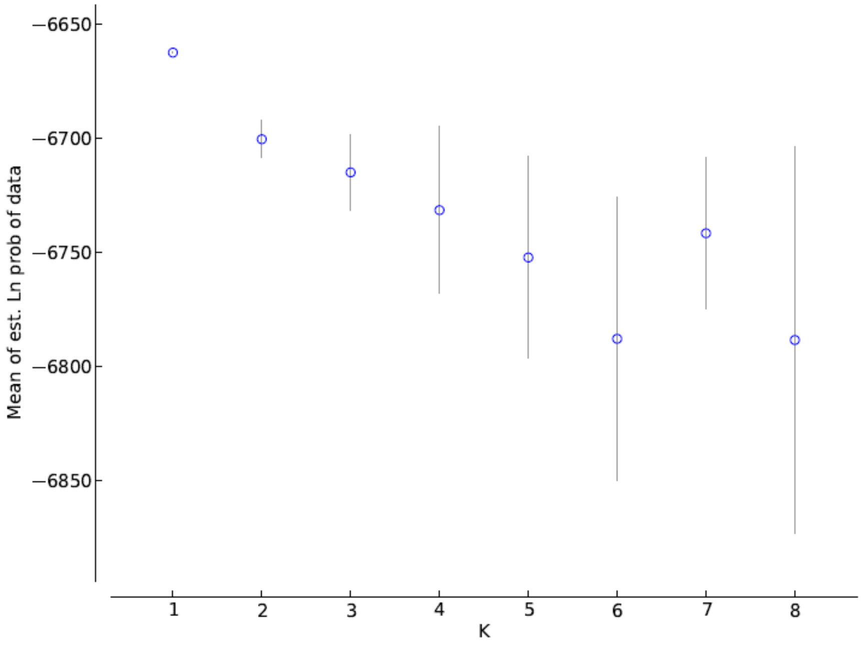

B

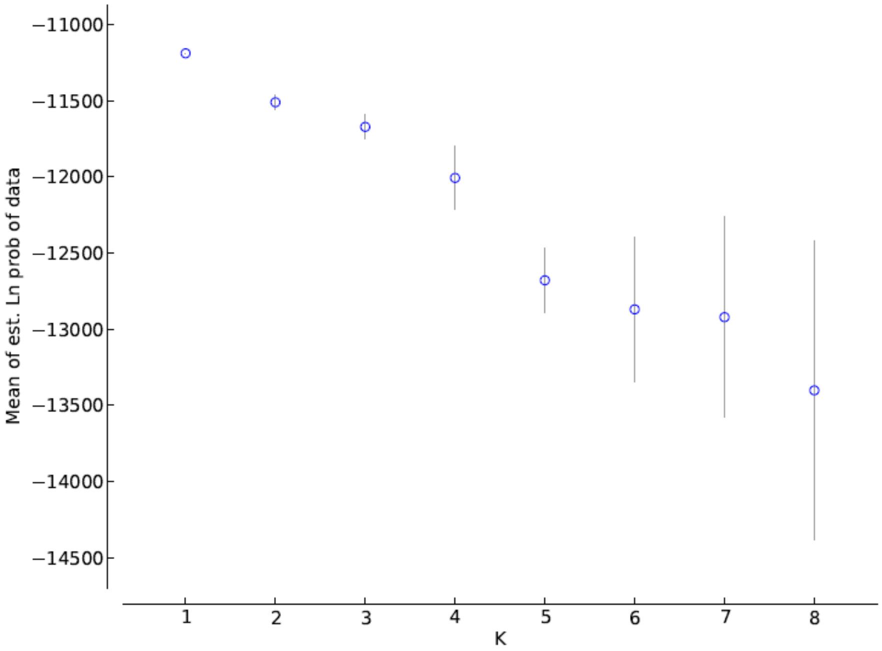

Supplement: Supplementary file 5 — Figure S3. Mean estimated Ln probability of data ± SD for K 1 through 8, averaged across 10 runs, for known and unknown-provenance falcons. A) Results of STRUCTURE analysis for 54 known-provenance chicks sampled from California’s San Francisco East Bay Area, Pinnacles National Park and the Mojave Desert that were genotyped at 123 SNP loci. B) Results of STRUCTURE analysis for a mix of 90 known and unknown provenance individuals sampled in California and Idaho and genotyped at 123 SNP loci. (PDF 268 kb) [file 12864_2018_4615_MOESM5_ESM.pdf]
