# Supplementary material for: New insights into the phylogenetics and population structure of the prairie falcon (Falco mexicanus)
Source: BMC Genomics. 2018 Apr 4;19:233. doi: 10.1186/s12864-018-4615-z (PMC5885362; doi:10.1186/s12864-018-4615-z)

Additional file 6: Supplementary Figure 4. Results of LOSITAN and BAYESCAN analyses. a) The confidence area for candidate loci under positive selection is shown in red. LOSITAN identified two outlier loci: X1613239_349604 (CACNA1G) and X1613580_160905 (A2ML1). b) No outlier loci were detected via BAYESCAN analysis. Posterior odds are plotted on the x axis and F_{ST} index values on the y axis.

A

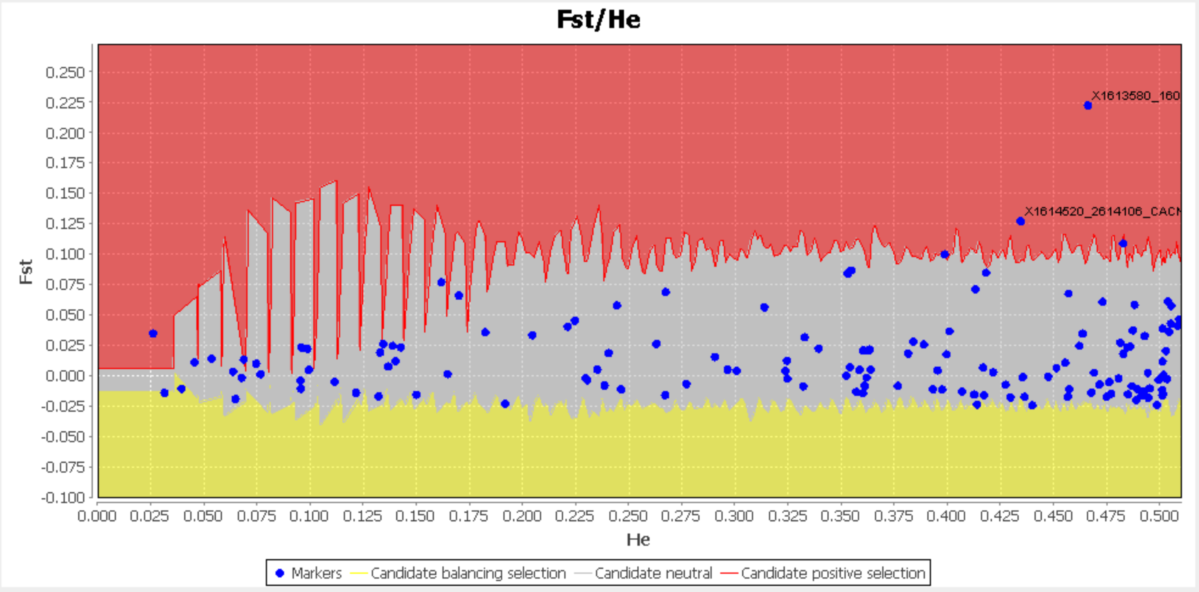

B

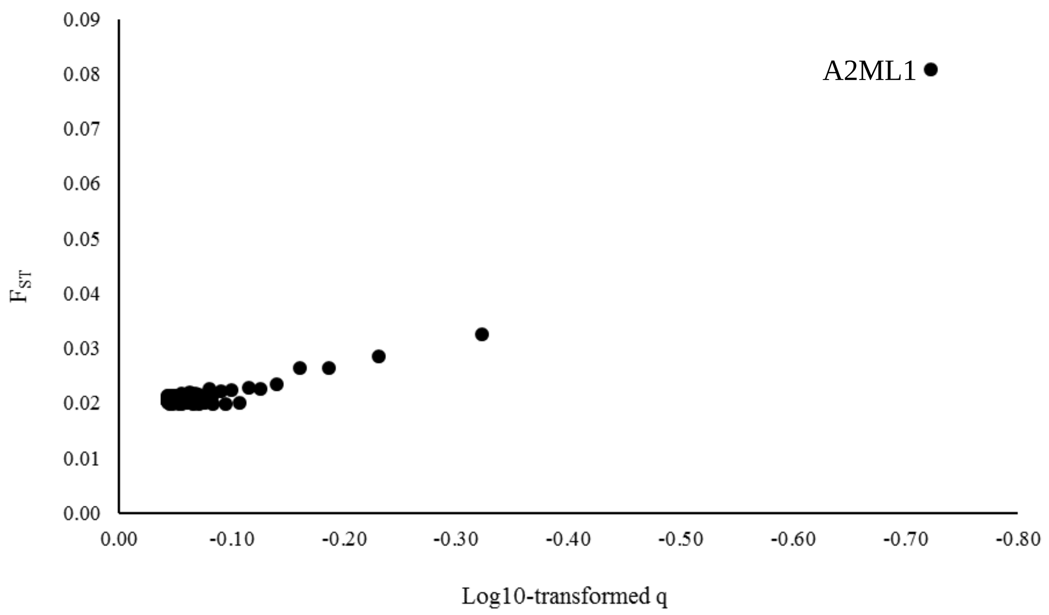

Supplement: Supplementary file 6 — Figure S4. Results of LOSITAN and BAYESCAN analyses. a) The confidence area for candidate loci under positive selection is shown in red. LOSITAN identified two outlier loci: X1613239_349604 (CACNA1G) and X1613580_160905 (A2ML1). b) No outlier loci were detected via BAYESCAN analysis. Posterior odds are plotted on the x axis and FST index values on the y axis. (PDF 36 kb) [file 12864_2018_4615_MOESM6_ESM.pdf]
