# Supplementary material for: New insights into the phylogenetics and population structure of the prairie falcon (Falco mexicanus)
Source: BMC Genomics. 2018 Apr 4;19:233. doi: 10.1186/s12864-018-4615-z (PMC5885362; doi:10.1186/s12864-018-4615-z)
Supplement: Supplementary file 7 — Table S3. Locus-specific pairwise FST values for prairie falcons in the San Francisco East Bay Area (East Bay), Pinnacles National Park (Pinnacles), the Mojave Desert (Mojave) and Idaho. (PDF 281 kb) [file 12864_2018_4615_MOESM7_ESM.pdf]

Additional file 7: Supplementary Table 3. Locus-specific pairwise F_{ST} values for prairie falcons in the San Francisco East Bay Area (East Bay), Pinnacles National Park (Pinnacles), the Mojave Desert (Mojave) and Idaho.

Locus	Gene	Pairwise F_{ST} values					
		East Bay/Idaho	East Bay/Mojave	East Bay/Pinnacles	Idaho/Mojave	Idaho/Pinnacles	Mojave/Pinnacles
X1606324_54556	MAP2	0.02	0.02	0.01	0.00	0.03	0.00
X1611790_179073	RECQL4	0.02	0.01	0.01	0.03	0.02	0.02
X1611802_3436465	FAN1	0.01	0.00	0.02	0.05	0.01	0.00
X1611802_3461010	MPHOSPH10	0.16	0.11	0.08	0.04	0.01	0.02
X1611828_3063350	LOC102058362	0.02	0.02	0.01	0.03	0.04	0.01
X1611951_4700267	KDR	0.03	0.00	0.00	0.08	0.06	0.03
X1612026_2960740	COL4A5	0.00	0.00	0.05	0.03	0.01	0.01
X1612041_807987	SLC4A1	0.10	0.02	0.01	0.13	0.16	0.03
X1612096_1127622	DAGLA	0.03	0.02	0.02	0.04	0.01	0.02
X1612100_28719	TLK2	0.02	0.05	0.01	0.19	0.01	0.11
X1612142_1217548	TLR5	0.01	0.02	0.02	0.01	0.01	0.02
X1612142_986460	TP53BP2	0.02	0.02	0.02	0.01	0.03	0.02
X1612176_151147	LOC101912977	0.18	0.03	0.01	0.03	0.11	0.00
X1612196_132010	RBM5	0.06	0.02	0.01	0.03	0.12	0.01
X1612201_2037184	AMER3	0.00	0.05	0.04	0.02	0.02	0.02
X1612204_3995128	LOC105403708	0.30	0.27	0.15	0.02	0.00	0.00
X1612204_4671637	ABC1	0.38	0.15	0.20	0.05	0.00	0.01
X1612233_1395268	LOC102056891	0.00	0.02	0.01	0.01	0.00	0.02
X1612346_10672156	NPAS2	0.00	0.01	0.02	0.02	0.01	0.01

X1612346_15212015	F10	0.02	0.00	0.01	0.03	0.03	0.01
X1612432_441003	CCDC129	0.02	0.00	0.01	0.02	0.06	0.03
X1612441_3381811	PDE1A	0.14	0.01	0.02	0.20	0.14	0.01
X1612469_1301514	ATXN1	0.06	0.01	0.01	0.01	0.10	0.02
X1612469_5271128	KIAA0319	0.02	0.01	0.02	0.00	0.02	0.00
X1612498_2735579	OBSCN	0.01	0.00	0.01	0.03	0.02	0.02
X1612498_64209	KRBA1	0.02	0.01	0.09	0.03	0.01	0.04
X1612503_1787024	TNNI3K	0.01	0.03	0.04	0.02	0.02	0.02
X1612503_840990	LRRC7	0.01	0.02	0.02	0.01	0.01	0.02
X1612520_570674	WIF1	0.09	0.01	0.01	0.03	0.05	0.02
X1612520_6017799	CNTN1	0.01	0.02	0.01	0.01	0.02	0.01
X1612536_143341	SHANK3	0.03	0.02	0.13	0.04	0.13	0.13
X1612555_1332412	DZIP1L	0.01	0.05	0.01	0.01	0.03	0.00
X1612562_4406105	VWA3A	0.00	0.03	0.02	0.01	0.01	0.03
X1612562_4811621	AIMP2	0.03	0.03	0.01	0.04	0.01	0.01
X1612563_323918	PCNT	0.02	0.02	0.01	0.05	0.02	0.05
X1612628_802176	SUCNR1	0.05	0.01	0.01	0.01	0.05	0.01
X1612632_1214371	MYH10	0.10	0.02	0.00	0.05	0.20	0.00
X1612643_3216746	FAM98B	0.00	0.01	0.02	0.02	--	0.06
X1612666_3119752	ADAM19	0.01	0.01	0.05	0.03	0.01	0.02
X1612666_6324471	MAPK9	0.01	0.01	0.09	0.05	0.14	0.02
X1612677_3028174	A4	0.02	0.02	0.00	0.03	0.03	0.02
X1612692_388076	LOC102054152	0.02	0.04	0.01	0.07	0.00	0.01
X1612694_3983000	FAH	0.01	0.01	0.01	0.04	0.01	0.01
X1612694_4470385	FANCI	0.12	0.04	0.00	0.01	0.03	0.01
X1612694_9639101	LRRK1	0.01	0.02	0.01	0.01	0.05	0.01
X1612734_62494	LOC102057144	0.00	0.00	0.01	0.05	0.01	0.02
X1612792_419173	STARD9	0.01	0.00	0.00	0.03	0.04	0.02
X1612806_1059367	LOC102058563	0.03	0.01	0.05	0.01	0.08	0.16
X1612827_362597	SLC6A19	0.04	0.02	0.02	0.02	0.02	0.03

X1612842_9736	WDR1	0.02	0.03	0.02	0.01	0.03	0.01
X1612844_2076748	TGM4	0.11	0.01	0.02	0.02	0.08	0.00
X1612845_1604447	MAOB	0.00	0.02	0.01	0.02	0.01	0.02
X1612849_22781	LOC102051644	0.02	0.00	0.01	0.00	0.01	0.02
X1612849_6445227	RCN1	0.02	0.02	0.02	0.02	0.04	0.00
X1612849_741062	SHANK2	0.01	0.02	0.03	0.02	--	0.06
X1612865_649251	GTPBP10	0.01	0.01	0.01	0.03	0.04	0.03
X1612932_487927	GRIA2	0.02	0.02	0.00	0.02	0.00	0.01
X1612946_1778604	CTTNBP2	0.04	0.06	0.03	0.04	0.03	0.02
X1612964_1456785	ECM2	0.05	0.02	0.01	0.06	0.02	0.01
X1612964_2693519	HRH1	0.03	0.00	0.00	0.03	0.11	0.07
X1612966_512205	TBC1D22A	0.02	0.03	0.02	0.03	0.04	0.02
X1612996_1597995	CEP44	0.02	0.00	0.01	0.03	0.03	0.02
X1613054_468852	G6PC	0.02	0.00	0.01	0.03	0.03	0.03
X1613056_1192015	PIGW	0.10	0.01	0.02	0.02	0.09	0.01
X1613057_67937	EVI2B	0.09	0.02	0.01	0.12	0.03	0.00
X1613088_104454	SH2D3C	0.03	0.03	0.00	0.04	0.00	0.03
X1613100_340329	LOC101913854	0.01	0.01	0.01	0.03	0.02	0.03
X1613106_1290319	CTSE	0.06	0.01	0.00	0.18	0.13	0.02
X1613106_169908	CHIA	0.00	0.05	0.01	0.01	0.01	0.03
X1613140_341712	PRSS12	0.14	0.06	0.02	0.02	0.06	0.01
X1613141_790350	FRMPD4	0.03	0.03	0.00	0.02	0.04	0.01
X1613177_217831	LOC101916644	0.01	0.14	0.09	0.04	0.00	0.01
X1613179_1746207	MMRN1	0.02	0.04	0.00	0.01	0.03	0.10
X1613179_594551	RPS3A	0.01	0.04	0.00	0.11	0.03	0.00
X1613239_349604	CNOT1	0.02	0.02	0.02	0.02	0.03	0.02
X1613259_1551290	LOC104696352	0.02	0.03	0.02	0.03	0.02	0.03
X1613262_3560666	GPR126	0.03	0.01	0.01	0.00	0.01	0.02
X1613274_21823	OPN3	0.02	0.02	0.01	0.01	0.01	0.02
X1613297_125040	CRB1	0.03	0.02	0.01	0.00	0.03	0.02

X1613300_47379	LOC101924349	0.01	0.01	0.01	0.03	0.02	0.02
X1613314_1496853	SMC1B	0.00	0.03	0.02	0.03	0.09	0.14
X1613332_33046	VPS37D	0.02	0.01	0.01	0.05	0.03	0.02
X1613342_8099358	RSPO4	0.01	0.03	0.03	0.01	0.03	0.07
X1613342_9318881	ZNFX1	0.01	0.02	0.02	0.02	0.07	0.01
X1613381_334429	SLC26A3	0.13	0.12	0.03	0.03	0.01	0.01
X1613384_1458567	CACNA1D	0.08	0.03	0.00	0.07	0.19	0.00
X1613408_287251	NLGN3	0.01	0.02	0.02	0.02	0.01	0.01
X1613408_431584	TEX11	0.01	0.01	0.01	0.00	0.00	0.02
X1613423_3288843	LAMA1	0.02	0.01	0.01	0.06	0.02	0.02
X1613444_602139	LOC101922066	0.03	0.03	0.01	0.03	0.02	0.01
X1613464_149043	BRIGHT.like	0.00	0.01	0.01	0.04	0.02	0.02
X1613464_1822371	SPG11	0.04	0.01	0.02	0.02	0.04	0.00
X1613481_407298	PNRC1	0.03	0.01	0.02	0.04	0.03	0.02
X1613501_2323466	RNF17	0.02	0.02	0.01	0.00	0.01	0.02
X1613514_1252656	WNK1	0.02	0.01	0.03	0.06	0.03	0.08
X1613518_109196	PLEKHA8	0.03	0.01	0.01	0.01	0.00	0.02
X1613522_786598	EFHB	0.12	0.04	0.01	0.01	0.06	0.01
X1613531_179158	SLC4A7	0.09	0.06	0.06	0.03	0.03	0.02
X1613536_116023	RELT	0.03	0.00	0.02	0.01	0.02	0.00
X1613547_2761141	GEF	0.12	0.00	0.02	0.02	0.01	0.02
X1613571_240446	NCKAP5	0.02	0.01	0.01	0.01	0.01	0.02
**X1613580_160905**	**A2ML1**	**0.41**	**0.16**	**0.03**	**0.03**	**0.44**	**0.19**
X1613580_29403	LOC102048449	0.03	0.02	0.02	0.04	0.04	0.03
X1613580_5715670	GSK3B	0.03	0.03	0.02	0.02	0.01	0.03
X1613596_4590629	ROBO2	0.01	0.02	0.01	0.02	0.02	0.03
X1613622_3304304	DNAH8	0.02	0.07	0.08	0.02	0.12	0.29
X1613623_1215243	RIPK2	0.02	0.01	0.01	0.02	0.00	0.01
X1613637_203205	LOC101919368	0.03	0.14	0.04	0.01	0.02	0.01
X1613640_779706	ANO10	0.04	0.02	0.01	0.02	0.07	0.05

X1613661_3074346	TNMD	0.00	0.02	0.20	0.04	0.08	0.06
X1613665_1751956	LOC103924686	0.06	0.03	0.04	0.03	0.02	0.03
X1613666_3047902	TEX2	0.01	0.02	0.01	0.02	0.02	0.00
X1613669_4864784	NID1	0.03	0.02	0.02	0.02	0.16	0.06
X1613669_6633260	FMN2	0.01	0.03	0.01	0.01	0.06	0.00
X1613695_1259234	SMOC2	0.03	0.02	0.01	0.04	0.03	0.02
X1613751_1071374	SORBS2	0.02	0.27	0.05	0.11	0.02	0.09
X1613751_2247454	ANKRD50	0.02	0.11	0.12	0.00	0.00	0.03
X1613785_129109	DNAJC6	0.02	0.01	0.02	0.04	0.02	0.02
X1613897_10629663	C8B	0.02	0.02	0.04	0.01	0.06	0.14
X1613897_3358312	PTPRF	0.14	0.02	0.04	0.00	0.00	0.02
X1613897_9109052	LRP8	0.01	0.07	0.01	0.01	0.05	0.17
X1614105_247653	ASB14	0.01	0.03	0.01	0.13	0.01	0.05
X1614105_566001	ABHD6	0.12	0.02	0.02	0.00	0.09	0.00
X1614105_5966489	ADAMTS9	0.01	0.01	0.03	0.03	0.01	0.01
X1614131_3786385	ARHGEF	0.02	0.02	0.02	0.04	0.02	0.01
X1614131_6229234	ERCC5	0.02	0.06	0.03	0.00	0.08	0.20
X1614264_2224382	SMO	0.05	0.12	0.01	0.03	0.02	0.01
X1614282_1141302	EML4	0.02	0.01	0.02	0.01	0.02	0.00
X1614298_1387066	PLA2G6	--	--	0.04	--	0.01	0.02
X1614305_2995114	TBC1D1	0.21	0.04	0.02	0.03	0.06	0.02
X1614314_3008240	LRRC16A	0.02	0.02	0.00	0.03	0.03	0.01
X1614362_1546835	TSNARE1	0.00	0.07	0.03	0.00	0.02	0.01
X1614474_953448	ST6GALNAC1	0.03	0.06	0.07	0.04	0.03	0.03
X1614506_145763	LARP7	0.01	0.02	0.01	0.03	0.01	0.02
X1614520_2614106	CACNA1G	0.27	0.19	0.11	0.02	0.03	0.01
X1614520_3065971	USH1G	0.02	0.02	0.00	0.03	0.02	0.02
X1614662_684404	PARPBP	0.03	0.06	0.02	0.06	0.01	0.01
X1614686_5293396	ACADS	0.02	0.03	0.01	0.00	0.03	0.00
X1614688_1973115	IL1RAP	0.04	0.02	0.01	0.07	0.07	0.02

X1615221_81774	POU1F1	0.02	0.09	0.02	0.00	0.00	0.06
X1615277_1808636	PAPSS2	0.02	0.02	0.01	0.03	0.02	0.01
X1615281_1321851	AGA	0.00	0.02	0.01	0.01	0.01	0.02
X1615288_3118567	FLII	0.01	0.07	0.02	0.17	0.00	0.06
